# Risk of cardiovascular disease among cancer survivors: Protocol of a pooled analysis of population-based cohort studies

**DOI:** 10.3389/fcvm.2022.926218

**Published:** 2022-08-05

**Authors:** Botao Yu, Zubing Mei, Hang Yu, Yan Wang, Qian Geng, Jin Pu

**Affiliations:** ^1^Emergency Department, The Second Affiliated Hospital of Medical College of Qingdao University, Qingdao, China; ^2^Anorectal Disease Institute of Shuguang Hospital, Shanghai, China; ^3^Department of Anorectal Surgery, Shuguang Hospital, Shanghai University of Traditional Chinese Medicine, Shanghai, China; ^4^Emergency Department, Changhai Hospital, Naval Military Medical University, Shanghai, China; ^5^The Second Department of Neurology, The Third Affiliated Hospital of Xinxiang Medical University, Xinxiang, China; ^6^Special Clinic of Changhai Hospital, Naval Military Medical University, Shanghai, China

**Keywords:** cardiovascular disease, cancer, population-based cohort study, risk, pooled analysis

## Abstract

**Introduction:**

Cancer and cardiovascular disease remain leading causes of death and disability worldwide, which places a heavy burden on public health systems and causes widespread suffering. Because these entities have highly overlapping risk factors, including hyperlipidemia, hypertension, diabetes, obesity, smoking and other lifestyle factors, many studies have reported that they have similar etiological mechanisms. Accumulating evidence indicates that there is an increased risk of cardiovascular disease among cancer survivors compared with the general population. However, whether cancer is associated with an increased risk of cardiovascular disease remains controversial.

**Methods and analysis:**

We will conduct and report the meta-analysis strictly based on the Cochrane Handbook for Systematic Reviews and the Meta-analysis of Observational Studies in Epidemiology guidelines combined with the Preferred Reporting Items for Systematic Reviews and Meta-analysis for Protocols (PRISM-P). This meta-analysis was registered with PROSPERO (registration number CRD42022307056). We will search for studies published from database inception to December 1, 2021, regardless of language or date, in three electronic databases (PubMed, EMBASE, and Cochrane Library) to identify and appraise cohort studies examining the relationship between cancer and subsequent cardiovascular disease risk. The literature screening, inclusion and data extraction will be conducted independently by two investigators using pre-designed standardized data extraction forms. A senior investigator will be consulted in cases of disagreement. We will assess risk of bias in the included cohort studies using the Newcastle–Ottawa Scale (NOS). Quantitative synthesis will be conducted using a random-effects model. To explore potential sources of heterogeneity, we will carry out multiple sensitivity analysis, meta-regression and subgroup analysis according to baseline characteristics. Publication bias will be evaluated through visual inspection of funnel plot asymmetry as well as by Begg's rank correlation test and Egger's weighted linear regression test.

## Introduction

Cancer and cardiovascular disease remain the leading causes of death and disability worldwide, which places a heavy burden on public health systems and causes widespread suffering (1–6). It has been reported that approximately 18 million people worldwide are diagnosed with cancer each year (7). In the last two decades, the survival rate of cancers has improved with the continuous development of early diagnosis and improvement of treatment modalities (8–11). Indeed, more than half of individuals in high-income groups are expected to survive for 10 years or more (12).

Cancer and cardiovascular disease have highly overlapping risk factors, including hyperlipidemia, hypertension, diabetes, obesity, smoking and other lifestyle factors (13–15), and many studies have reported that they share similar etiological mechanisms (16–18). Nevertheless, whether cancer is associated with an increased risk of cardiovascular disease has been controversial. Several studies have demonstrated that people diagnosed with cancer, such as breast cancer, lung cancer and hematological malignancies, have a higher risk of cardiovascular disease than the general population (12, 19–30). Other studies, however, have found that cancer survivors have a lower risk of cardiovascular disease, including those with gastric cancer (31). Therefore, these conflicting results prompted us to conduct a systematic comprehensive analysis to assess the relationship between cancer and subsequent cardiovascular disease risk. For the reasons given above, we will carry out a meta-analysis to evaluate the risk of cardiovascular disease among cancer survivors based on high-quality population-based cohort studies.

## Methods and analysis

### Protocol registration

We will conduct and report our meta-analysis strictly based on the Cochrane Handbook for Systematic Reviews and the Meta-analysis of Observational Studies in Epidemiology guidelines combined with the Preferred Reporting Items for Systematic Reviews and Meta-analysis for Protocols (PRISM-P) (32) (Supplementary Table S1). Details of the meta-analysis protocol, which has been registered with the registration number CRD42022307056, can be found at the PROSPERO website (www.crd.york.ac.uk/prospero).

### Data sources and search strategies

We will search for studies published from database inception to December 1, 2021, regardless of language or date, in three commonly used electronic databases, namely, PubMed, EMBASE, and Cochrane Library, to identify and appraise cohort studies examining the relationship between cancer and subsequent cardiovascular disease risk. We will use Medical Subject Heading (MeSH) or EMBASE Subject Heading (Emtree) terms along with free-text words that are associated with cancer and cardiovascular disease. The following search keywords and search logic will include cancer/tumor/tumor/oncology/neoplasm^*^/malignanc^*^/carcinoma AND cardiovascular diseases/coronary disease/stroke/myocardial infarction/myocardial ischemia/heart failure AND cohort study/longitudinal study/follow-up study/prospective study/retrospective study (PubMed search strategy in Table 1). We will also identify additional relevant studies by reviewing reference lists from the articles identified and a manual search of abstracts from annual meetings of the European Society for Medical Oncology (ESMO) and the American Society of Clinical Oncology (ASCO). When there are multiple studies describing the same cohort, the study involving the largest number of participants or the most recent sample cohort will be included. Duplicate citations will first be automatically removed from the initial database searches using EndNote version X9 (Thomson Reuters), after which additional duplicate citations will be manually removed by comparing authors, article titles and publication dates. We will carry out these selection processes separately by two investigators. Conflicts will be handled by discussion, and a senior investigator will be consulted if necessary.

**Table 1:** Search strategy for the Pubmed database.

**Search strategy**
**Cancer terms**
1 “Neoplasms”[Mesh]
2 (cancer* or oncology* or tumor* or tumour* or neoplas* or malignan* or carcinoma* or adenocarcinoma* or choriocarcinoma* or leukemia* or leukaemia* or metastat* or sarcoma* or teratoma* or lymphoma* or melanoma* or sarcoma*) [Title/Abstract]
3 or/1–2
**Cardiovascular disease terms**
4 “Cardiovascular Diseases”[MeSH]
5 (Cardiovascular Disease OR Disease, Cardiovascular OR Diseases, Cardiovascular OR major adverse cardiovascular event)[Title/Abstract]
6 “Coronary Disease”[MeSH]
7 (Coronary Diseases OR Disease, Coronary OR Diseases, Coronary OR Coronary Heart Disease OR Coronary Heart Diseases OR Heart Disease, Coronary OR Heart Diseases, Coronary OR coronary artery disease)[Title/Abstract]
8 “Stroke”[MeSH]
9 (Strokes OR Cerebrovascular Accident OR Cerebrovascular Accidents OR Vascular Accident, Brain OR Brain Vascular Accident OR Brain Vascular Accidents OR Vascular Accidents, Brain OR Apoplexy)[Title/Abstract]
10 “Myocardial Infarction”[MeSH]
11 (Infarction, Myocardial OR Infarctions, Myocardial OR Myocardial Infarctions OR Cardiovascular Stroke OR Cardiovascular Strokes OR Stroke, Cardiovascular OR Strokes, Cardiovascular OR Myocardial Infarct OR Infarct, Myocardial OR Infarcts, Myocardial OR Myocardial Infarcts OR Heart Attack OR Heart Attacks OR heart infarction OR heart infarct)[Title/Abstract]
12 “Myocardial Ischemia”[MeSH]
13 (Ischemia, Myocardial OR Ischemias, Myocardial OR Myocardial Ischemias OR Ischemic Heart Disease OR Heart Disease, Ischemic OR Disease, Ischemic Heart OR Diseases, Ischemic Heart OR Heart Diseases, Ischemic OR Ischemic Heart Diseases)[Title/Abstract]
14 “Heart Failure”[MeSH]
15 (Cardiac Failure OR Myocardial Failure)[Title/Abstract]
16 or/4-15
**Study design terms**
17 “Retrospective studies”[Mesh]
18 “Cohort studies”[Mesh]
19 “Longitudinal studies”[Mesh]
20 “Follow-up studies”[Mesh]
21 “Prospective studies”[Mesh]
22 (cohort or longitudinal or followup or prospective*or retrospective* or database* or population* or follow up)[Title/Abstract]
23 “Registries”[Mesh]
24 (registry or registries) [Title/Abstract]
25 or/17–24
**Final search results: Combining cancer and stroke and the study design**
26 3 and 16 and 25

### Eligibility criteria

We will include studies with the following inclusion criteria in qualitative analyses: (1) studies involving a prospective or retrospective population-based cohort; (2) participants diagnosed with cancer but no history of cardiovascular disease or not diagnosed with cardiovascular disease at the time of participant enrolment based on the International Classification of Diseases, Seventh to Tenth Revision (ICD 7–10) criteria in the population-based cohort and diagnosed with one type of cardiovascular disease during the subsequent follow-up period; (3) studies reporting risk estimates, including relative risk (RR), hazard ratio (HR) and odds ratio (OR) with 95% confidence intervals (CIs), or studies that can provide relevant data to be used to estimate risk ratios. We will exclude hospital-based cohort studies and those with inadequate data to estimate risk ratios.

### Data extraction

Data extraction will be conducted independently by two investigators using pre-designed standardized data extraction forms and crosschecked by a third investigator. Disagreements will be resolved by consensus. The following study characteristics will be abstracted: first author, publication year and design, geographic region, observation period, participant age at cancer diagnosis, comparison population, criteria of diagnosis for cancer and cardiovascular disease, study findings and risk estimates of the association of cardiovascular disease with cancer.

### Assessment of risk of bias

The risk of bias for the included studies will be assessed using the Newcastle–Ottawa scale (NOS) tool according to three major aspects: representativeness of the patients, ascertainment of exposure and outcomes and adequacy of follow-up (33). A total of nine scores will be assigned to nine item domains. Studies will be judged to be at low risk of bias (≥8 points) or moderate to high risk of bias (<8 points) (15).

### Statistical analysis

All statistical meta-analyses will be carried out using Stata software (version 12.0; Stata Corp LP, College Station, TX). The summary RR among cancer survivors compared with that among the non-cancer population accompanied by its 95% CI will be defined as the primary outcome. Given the expected large heterogeneity for the participants, study design, and clinical and methodological aspects among the included studies, we will adopt the DerSimonian–Laird random-effects model to calculate risk estimates (34). RR will be regarded as the common risk estimate for the association between cancer and cardiovascular disease risk. We will include RRs that are maximally adjusted for potential confounders for meta-analysis (15, 35). As fully adjusted models include the most hypothesized confounders, we will prioritize selecting fully adjusted risk estimates to meta-analyse RRs. Heterogeneity will be assessed by using the χ2 test on Cochran's Q statistic, and the *I*^2^ statistic will be employed to quantify the proportion of the total variation leading to heterogeneity, where an *I*^2^ > 50% indicates substantial heterogeneity (36).

We will further carry out subgroup analysis to explore potential sources of heterogeneity based on the following factors *post-hoc*: study design, geographic regions, methodological quality, outcome division, age, sex, attained age, adjuvant therapy, cancer site and cardiovascular disease type. In addition, sensitivity analysis will be carried out by removing one study at each time and repeating the analysis to evaluate whether any individual study considerably influences the summary estimates. Meta-regression will also be performed to explain the effects of heterogeneity. We will explore the possibility of publication bias with visual inspection of a funnel plot and with Begg's rank correlation test and Egger's weighted linear regression test if 10 or more studies are included in the meta-analysis (37, 38). To further assess the potential effects of publication bias, we will apply a Duvall and Tweedle trim-and-fill technique to estimate the number of studies with null effects that are missing from the meta-analysis (39). In all statistical analyses, *P* ≤ 0.05 will be considered statistically significant.

## Discussion

This study-level pooled analysis of all population-based cohort studies will provide a comprehensive overview and a quantitative summary of the risk of cardiovascular disease for cancer survivors compared with that for the general population. Moreover, we will explore the potential of heterogeneity by multiple subgroup analyses based on study baseline characteristics and examine the robustness of the results through sensitivity analysis.

This meta-analysis of population-based cohort studies will have several strengths. First, to the best of our knowledge, this study will be the first and most comprehensive one addressing such an association with highly representative populations. Second, we will strictly follow the Cochrane Handbook (40) to carry out the meta-analysis, and the risk of bias of all the included studies will be assessed using the NOS tool, which can provide clear knowledge of the study quality in our meta-analysis. Third, by conducting multiple subgroup, meta-regression and sensitivity analyses, we will thoroughly explore the sources of heterogeneity and robustness of the pooled estimates in this meta-analysis. Finally, a major study database search combined with manual search will be involved in our meta-analysis without language or date limitations, which can reduce the possibility of publication bias. Furthermore, if publication bias exists, the trim and fill method will be applied to adjust the risk estimates, which can provide reliable estimates for ‘missing' studies.

As a major limitation of this study, significant between-study heterogeneity is anticipated because we will include all types of cancer for analysis. Although multiple subgroup analyses and meta-regression will be conducted based on the available data from the included studies, some unknown confounding factors, such as cancer histology, cancer stage, performance status, and age of onset, will be highly diverse, making this study design clinically heterogeneous. However, to maximally control the impact of heterogeneity on the results, we will strictly adhere to the inclusion and exclusion criteria when we select suitable studies and use RRs maximally adjusted for potential confounders for meta-analysis and conducted multiple subgroup analyses to explore the potential sources of heterogeneity. Therefore, this study will to some extent provide us with a general risk estimate of cardiovascular disease among cancer survivors. The findings of this study will provide valuable clinical significance for the CVD risk of cancer survivors and the approximate risk estimate of CVD for cancer survivors. Overall, prevention and treatment of CVD during early treatment after cancer diagnosis have important clinical significance. Oncologists can choose cardiovascular protection treatment strategies according to the adjuvant treatment options and risk factors for cancer patients to reduce the organ function damage caused by treatment as much as possible.

## Ethics statement

Ethical review and approval was not required as this study would only use published data without involving human participants.

## Author contributions

Conceptualization: BY, ZM, HY, QG, and JP. Data curation, formal analysis, methodology, software, and writing–original draft: BY and ZM. Funding acquisition: BY. Investigation, resources, and visualization: BY, QG, and JP. Project administration and validation: BY, ZM, HY, YW, QG, and JP. Supervision: ZM, QG, and JP. Writing–review & editing: BY, YW, and ZM. All authors contributed to the article and approved the submitted version.

## Funding

This work was supported by Qingdao Key Health Discipline Development Fund (Grant No. PD-202102018).

## Conflict of interest

The authors declare that the research was conducted in the absence of any commercial or financial relationships that could be construed as a potential conflict of interest.

## Publisher's note

All claims expressed in this article are solely those of the authors and do not necessarily represent those of their affiliated organizations, or those of the publisher, the editors and the reviewers. Any product that may be evaluated in this article, or claim that may be made by its manufacturer, is not guaranteed or endorsed by the publisher.

## Abbreviations

ASCO: American Society of Clinical Oncology
CI: confidence interval
Emtree: EMBASE Subject Heading
ESMO: the European Society for Medical Oncology
HR: hazard ratio
ICD: International Classification of Diseases
MeSH: Medical Subject Heading
NOS: Newcastle–Ottawa scale
OR: odd ratio
PRISM-P: Preferred Reporting Items for Systematic Reviews and Meta-analysis for Protocols
RR: relative risk.
